# A Time-Variant Reliability Model for Copper Bending Pipe under Seawater-Active Corrosion Based on the Stochastic Degradation Process

**DOI:** 10.3390/ma11040507

**Published:** 2018-03-27

**Authors:** Bo Sun, Baopeng Liao, Mengmeng Li, Yi Ren, Qiang Feng, Dezhen Yang

**Affiliations:** School of Reliability and Systems Engineering, Beihang University, Beijing 100191, China; sunbo@buaa.edu.cn (B.S.); lbp2016@buaa.edu.cn (B.L.); 13051583907@163.com (M.L.); renyi@buaa.edu.cn (Y.R.); dezhenyang@buaa.edu.cn (D.Y.)

**Keywords:** time-variant reliability, stochastic degradation process, copper bending pipe, seawater-active corrosion

## Abstract

In the degradation process, the randomness and multiplicity of variables are difficult to describe by mathematical models. However, they are common in engineering and cannot be neglected, so it is necessary to study this issue in depth. In this paper, the copper bending pipe in seawater piping systems is taken as the analysis object, and the time-variant reliability is calculated by solving the interference of limit strength and maximum stress. We did degradation experiments and tensile experiments on copper material, and obtained the limit strength at each time. In addition, degradation experiments on copper bending pipe were done and the thickness at each time has been obtained, then the response of maximum stress was calculated by simulation. Further, with the help of one kind of Monte Carlo method we propose, the time-variant reliability of copper bending pipe was calculated based on the stochastic degradation process and interference theory. Compared with traditional methods and verified by maintenance records, the results show that the time-variant reliability model based on the stochastic degradation process proposed in this paper has better applicability in the reliability analysis, and it can be more convenient and accurate to predict the replacement cycle of copper bending pipe under seawater-active corrosion.

## 1. Introduction

Copper material with good corrosion resistance is widely used in a variety of harsh environments [1], but will still be affected by the inevitable seawater-active corrosion [2,3]. The copper bending pipe, which is applied in seawater piping systems, can change the direction of seawater flow. Thus, the impact of seawater-active corrosion and pressure on the copper bending pipe will lead to the degradation of the material properties, and then leakage will occur. Therefore, the time-variant reliability model of copper bending pipe under seawater-active corrosion is studied in order to provide a basis for improving the safety of seawater piping systems by reducing the failure caused by corrosion.

Due to the seawater-active corrosion, the performance of copper bending pipe is degraded [4,5], and the randomness and multiplicity of the degradation process under corrosion is complicated. Therefore, how to describe the degradation of copper bending pipe by a mathematical model is a difficult problem to be solved. The environment can adversely affect the mechanical behavior of material, and the copper bending pipe works in both the seawater environment and the atmospheric environment, simultaneously. Kang [6] studied the corrosion behaviors of metal and its degradation of fatigue life. Lin [7] and Hodgkiess [8] studied the corrosion behavior of copper material in seawater environments. Saha [9] studied the corrosion mechanism of copper material exposed in the atmosphere, and Sun [10] studied the stress corrosion of pipelines in seawater environments. Ossai [11] and Velazquez [12] studied the process of pipeline failure under corrosion environments. These studies show that seawater-active corrosion is the main reason for the failure of copper bending pipe, and the degradation of both material and thickness of pipeline are random. However, they only considered the effect of corrosion on the material, or the thickness of the pipeline, and did not address the problem by using a mathematical model to describe the stochastic degradation process. Actually, the reliability is influenced by the seawater-active corrosion of both material and pipeline, and these influences will change over time.

Therefore, the mathematical model of the stochastic degradation process and the time-variant reliability analysis method of copper bending pipe still need to be further studied. Song [13] proposed a stochastic method for analyzing the fuzzy systems. Vale [14] presented a stochastic model for characterizing the degradation process over time, but only one variable has been considered. Takano [15] studied the stochastic model of structure strength with geometrical imperfection and uncertainty in material property. Fang [16] and Bao [17], respectively, proposed a stochastic model updating, and solved it based on the Monte Carlo method. Deng [18] established a time-dependent degradation model, but did not consider the randomness of variables at each moment. Zhang [19] described the random effects of degradation modeling and remaining useful life, and the time-dependent functions were used to fit the degradation rules of characteristic parameters of random variables. In the analysis process of copper bending pipe, both the multiplicity and the randomness of variables should also be considered, and the Monte Carlo method may not be conducive to reducing the computation. Besides, in terms of reliability calculations, some scholars [20,21] have also studied the analysis method under conditions of variable randomization. When the time factor was considered, Li [22] solved the dynamic model with discrete theory, which gives us an idea for calculating the time-variant reliability. Jiang [23,24] proposed a stochastic process discretization method to calculate the time-variant reliability, but this method would reduce the accuracy of reliability due to the discrete stochastic process. Wang [25] translated the random processes and time parameters into random parameters to calculate the reliability at any time. This method is computationally simple but reduces the reliability accuracy. Therefore, it is necessary to propose a new method to solve the deficiencies of existing methods.

On the basis of these studies, in this paper, the characteristic parameters of variables in the stochastic degradation process at each time point will be extracted, and the degradation process will be described as a function of characteristic parameters over time. Combined with the operation condition of copper bending pipe, the time-variant reliability will be calculated by the interference theory. Then, after a comparison with the results of traditional methods and maintenance records, the accuracy of the method proposed in this paper will be verified. Finally, some conclusions will be raised for the whole paper.

## 2. Time-Variant Reliability Modeling Method

### 2.1. Failure Analysis of Copper Bending Pipe

**Figure 1:**
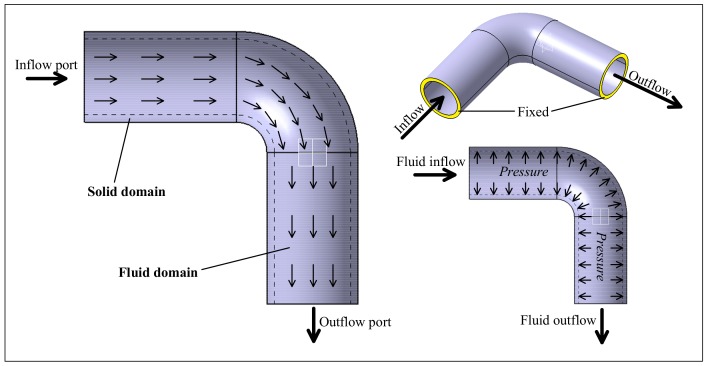
The structure and flow paths of copper bending pipe.

During the operation, the flow direction of seawater changes inside of the copper bending pipe. Figure 1 shows the structure and seawater flow paths of copper bending pipe. The flow of seawater flows from the inflow port to the outflow port, and the flow process brings a pressure to the inner wall of the copper bending pipe due to the presence of bending. Also, the copper bending pipe has imposed a fixed constraint (as shown in the Fixed mark in Figure 1) to ensure that it will not fall off the seawater pipeline system.

The copper bending pipe used in seawater piping systems is subject to seawater-active corrosion during its operation. The corrosion leads to a degradation of copper material in both mechanical properties and corrosion resistance, and results in a reduction in the limit strength of the copper material and a reduction in the thickness of the copper bending pipe. A lower thickness leads to a more severe stress concentration, which will increase the maximum stress of the copper bending pipe. When the maximum stress is greater than the limit strength, the copper bending pipe will fail. For other failure modes such as cracks, their probability of occurrence can be ignored according to the existing studies [26,27].

### 2.2. Stochastic Degradation Process Based on Modeling Method

In order to facilitate the study of time-variant reliability models for copper bending pipe, the following assumptions are made [28]:

(1) The thickness of the copper bending pipe is equal, regardless of the residual stresses during the forming process.
(2) Copper materials are assumed to be a material with uniform elastic properties; the Young’s modulus and Poisson’s ratio are not degraded.
(3) The fixed constraint of copper bending pipe is completely reliable. This means that the copper bending pipe will not fall off in any case.

On the basis of these assumptions, the stress–strength interference model was selected to model the reliability of copper bending pipe. In the process of work, both stress and strength of the copper bending pipe are random variables and degrade over time [29,30]. This process is defined as the stochastic degradation process. Based on the stochastic degradation process, the reliability model of copper bending pipe under seawater-active corrosion is shown in Equation (1).

(1) $$R\left(t\right)=P\{f\left(\sigma_{\lim}|a_{1}\left(t\right),a_{2}\left(t\right),\cdots ,a_{i}\left(t\right)\right)-f\left(\sigma_{s}^{\max}|b_{1}\left(t\right),b_{2}\left(t\right),\cdots ,b_{j}\left(t\right)\right)>0\}$$

where R(t) is the reliability of copper bending pipe at seawater-active corrosion time t. $\sigma_{lim}$ and $\sigma_{s}^{max}$ are separately the limit strength and the maximum stress of copper material and copper bending pipe. $f\left(\sigma_{lim}|a_{1}\left(t\right),a_{2}\left(t\right),\cdots ,a_{i}\left(t\right)\right)$ and $f\left(\sigma_{s}^{max}|b_{1}\left(t\right),b_{2}\left(t\right),\cdots ,b_{j}\left(t\right)\right)$ are the probability density function (PDF) of $\sigma_{lim}$ and $\sigma_{s}^{max}$ at seawater-active corrosion time t. $a_{1}\left(t\right),a_{2}\left(t\right),\cdots ,a_{i}\left(t\right)$ and $b_{1}\left(t\right),b_{2}\left(t\right),\cdots ,b_{j}\left(t\right)$ are the characteristic parameters of PDFs in $\sigma_{lim}$ and $\sigma_{s}^{max}$ at seawater-active corrosion time t, respectively.

As shown in Equation (2), the PDF of limit strength and maximum stress can be obtained by fitting their experimental values at each time points $t_{1},t_{2},\cdots ,t_{n}$ during the stochastic degradation process (X is represented by the limit strength or the maximum stress).

(2) $$\left[\begin{array}{c} X_{1}\\ X_{2}\\ \vdots \\ X_{n} \end{array}\right]=\left[\begin{array}{cccc} x_{11} & x_{12} & \cdots & x_{1m}\\ x_{21} & x_{22} & \cdots & x_{2m}\\ \vdots & \vdots & \ddots & \vdots \\ x_{n1} & x_{n2} & \cdots & x_{nm} \end{array}\right]\sim \left[\begin{array}{c} f\left(X_{1}\right)\\ f\left(X_{2}\right)\\ \vdots \\ f\left(X_{n}\right) \end{array}\right]$$

where $X_{i}$ and $x_{i1},x_{i2},\cdots ,x_{im}$ are the random variables and their experimental values at time point $t_{i}$ (i=1, 2,⋯,n), and the symbol “~” indicates that the PDF of $X_{i}$ is $f\left(X_{i}\right)$, and $f\left(X_{i}\right)$ has:

(3) $$X_{i}\sim f\left(\xi_{1}\left(t_{i}\right);\xi_{2}\left(t_{i}\right);\cdots ;\xi_{k}\left(t_{i}\right)\right)\;i=1,2,\cdots ,n$$

where $\xi_{1}\left(t_{i}\right),\xi_{2}\left(t_{i}\right),\cdots ,\xi_{k}\left(t_{i}\right)$ are the k characteristic parameters of $X_{i}$.

Then, the degradation of these characteristic parameters are analyzed. As it is shown in Equation (4), fitting the characteristic parameters which were obtained in $t_{1},t_{2},\cdots ,t_{n}$, the function of each characteristic parameter over time can be obtained [31]. The random variable changes over time, X, as Equation (5) shows. Therefore, the stochastic degradation process of limit strength and maximum stress in Equation (1) can be obtained, and the time-variant reliability of copper bending pipe can be calculated after solving it.

(4) $$\left[\begin{array}{cccc} \xi_{1}\left(t_{1}\right) & \xi_{1}\left(t_{2}\right) & \cdots & \xi_{1}\left(t_{n}\right)\\ \xi_{2}\left(t_{1}\right) & \xi_{2}\left(t_{2}\right) & \cdots & \xi_{2}\left(t_{n}\right)\\ \vdots & \vdots & \ddots & \vdots \\ \xi_{k}\left(t_{1}\right) & \xi_{k}\left(t_{2}\right) & \cdots & \xi_{k}\left(t_{n}\right) \end{array}\right]\sim \left[\begin{array}{c} f_{1}\left(t\right)\\ f_{2}\left(t\right)\\ \vdots \\ f_{k}\left(t\right) \end{array}\right]$$

(5) $$X\left(t\right)\sim f\left[f_{1}\left(t\right),f_{2}\left(t\right),\cdots ,f_{k}\left(t\right)\right]$$

where $f_{i}\left(t\right)$ is a function of the i-th characteristic parameter over time (i=1, 2,⋯,k).

### 2.3. Solution for Reliability Model

The reliability model of copper bending pipe is composed of two PDFs with i and j characteristic parameters, and with time variability, while numerical methods to solve the interference of two PDFs will face an incredible amount of computation. Under this premise, a kind of Monte Carlo method has been proposed to solve this reliability model.

Take the time period T into a number of discrete-time points 0,∆t,2∆t,3∆t,⋯,T, and the reliability of copper bending pipe at each discrete-time point can be calculated. Then, a time-variant reliability curve can be obtained (the smaller ∆t is, the smoother the reliability curve will be).

First, s pseudorandom values of the two PDFs were generated at each discrete-time point, with the pseudorandom set of limit strength defined as $s_{1}$, and the pseudorandom set of maximum stress defined as $s_{2}$. Then, two numbers were separately randomly sampled from $s_{1}$ and $s_{2}$, and their magnitude compared. A total of n samples and comparisons were made, and the number of times k that the number from $s_{1}$ was greater than $s_{2}$ was recorded. Finally, the reliability could be calculated by Equation (6) [32].

(6) $$R\left(t\right)=\frac{k\left(t\right)}{n\left(t\right)}\;t=0,\Delta t,2\Delta t,3\Delta t,\cdots ,T$$

where k(t), n(t), and R(t) are, respectively, the number of times that limit strength is greater than maximum stress, the number of samples and comparisons, and the reliability of copper bending pipe in discrete-time point t.

## 3. Experiments and Reliability Analysis

The degradation process of limit strength and thickness can be obtained by experiments, and then the mathematical relationship between thickness and maximum stress can be established by simulation. Therefore, this section reports the degradation process of limit strength and maximum stress by experiments and simulations, and the time-variant reliability of copper bending pipe will be calculated based on the reliability model.

### 3.1. Experiment Setup

Under seawater-active corrosion, the chemical or electrochemical reactions occur between the surface of copper material and seawater, which in turn leads to strength loss (limit strength reduced) and mass loss (thickness reduced). Therefore, based on the phenomenon of seawater-active corrosion, the corrosion of copper bending pipe was quantitatively measured by the experiment.

**Figure 2:**
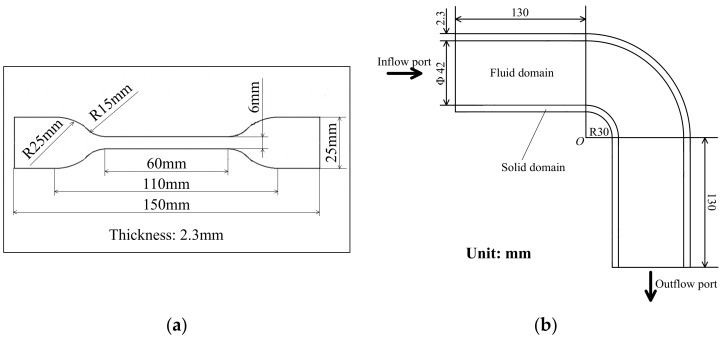
The dimensions of experiment sample: (**a**) the dimension of copper sample; (**b**) the dimension of copper bending pipe.

The experiment was carried out under the conditions shown in Table 1, and the dimensions of the copper bending pipe and copper material sample are shown in Figure 2 (the copper bending pipe was made of B10 copper alloy). In this case, the experiment period was 24 months and conducted at time intervals of 3 months to measure the PDFs of strength and thickness. There were three kinds of seawater velocity: 3 m/s, 6 m/s, and 9 m/s; the reliability analysis was carried out at a seawater velocity of 3 m/s and then extended to 6 m/s and 9 m/s to verify the above method.

Take the seawater velocity of 3 m/s as an example. During the experiment, the copper material samples were immersed in flowing seawater and their mechanical properties degraded due to seawater-active corrosion. The copper bending pipe, with seawater flowing inside, was exposed to the atmosphere. A certain number of samples was taken every three months. The tensile strength of the copper material samples and the thickness of the copper bending pipe were measured, and the data were recorded until the end of the experiment at 24 months.

### 3.2. Experiment Results

The experiment specimens were corroded to varying degrees after the corrosion experiments, and the tensile properties and thickness of these specimens were measured separately. The experiment data were recorded and experiments were repeated every three months.

There are 8 sets of tensile strength data and 8 sets of thickness data. Gamma distribution was selected to fit the limit strength of copper material samples at each seawater-active corrosion time in this paper [33,34]. After that, the experiment results (Appendix A) were fitted by Gamma distribution and the characteristic parameters at each seawater-active corrosion time are shown in Table 2. Then, according to Equation (4) and Equation (5), the limit strength of copper material is shown in Equation (7).

(7) $$\begin{array}{l} \alpha_{1}\left(t\right)=-2.2132t+472.43\;R^{2}=0.96\\ \beta_{1}\left(t\right)=0.0011t+0.2592\;R^{2}=0.94 \end{array}$$

where $\alpha_{1}$ and $\beta_{1}$ are the characteristic parameters of Gamma distribution at seawater velocity of 3 m/s.

The normal distribution has been selected to fit the thickness of the copper bending pipe [30]. After that, as shown in Appendix A, the characteristic parameters at each seawater-active corrosion time are shown in Table 3 (where μ and $\sigma^{2}$ are the characteristic parameters of normal distribution).

### 3.3. Stress Simulation Analysis

**Figure 3:**
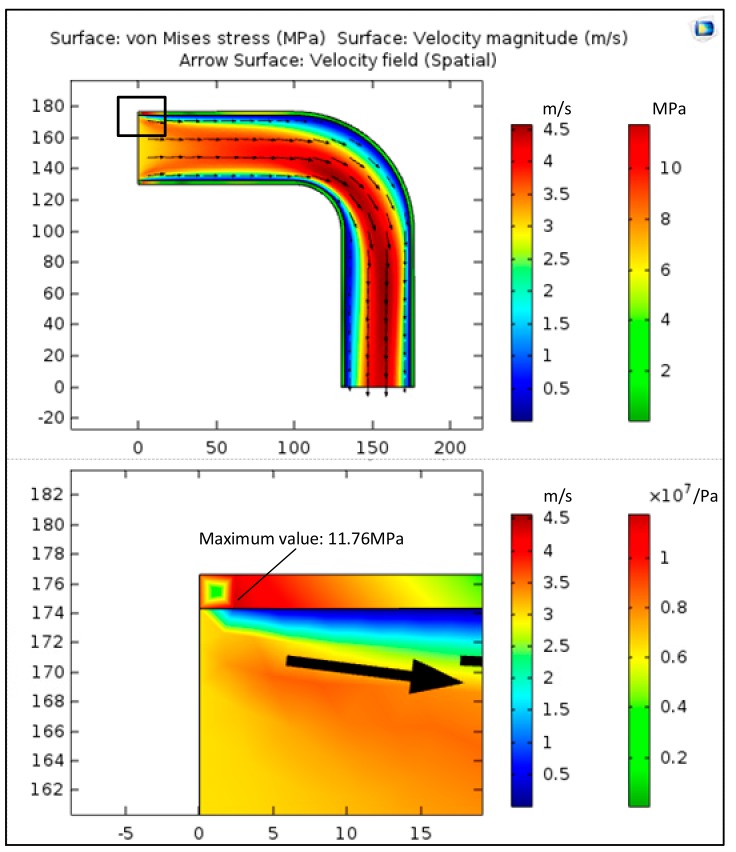
The stress field of copper bending pipe in initial state.

Due to the complexity of fluid-structure interaction, the mathematical relationship between thickness and maximum stress needs to be calculated by the finite element method. In the initial state, the thickness of the copper bending pipe was 2.3 mm. According to the working conditions of copper bending pipe, the fluid-structure interaction was the physics interface of the analytical model, and the boundary conditions of inlet velocity and outlet pressure has been chosen. Choosing the compiled equations with stationary, the stress field of copper bending pipe is calculated by COMSOL (The COMSOL Inc., Stockholm, Sweden), and as shown in Figure 3, it can be seen that the maximum stress is 11.76 MPa.

Then, based on the PDF in Table 3, generating lots of plots and cycle calling COMSOL for solving, the response of the maximum stress to the thickness can be obtained. After that, it can be seen that the maximum stress of copper bending pipe is subject to a logarithmic normal distribution, and the characteristic parameters at each time are shown in Table 4. The function that fits the maximum stress over time is shown in Equation (8).

(8) $$\ln\sigma_{s1}^{\max}\left(t\right)\sim N\left(0.0023t+2.407,\left(0.298t+0.1122\right)\times 10^{-3}\right)\;R_{\mu }^{2}=0.98,R_{\sigma^{2}}^{2}=0.99$$

### 3.4. Reliability Analysis

At this time, the time-variant model of limit strength and maximum stress under a seawater velocity of 3 m/s can be obtained. In the same way, the time-variant models under 6 m/s and 9 m/s are shown in Equation (9).

(9) $$\begin{array}{l} v_{2}=6\;m/s\;\begin{array}{l} \sigma_{\lim2}\sim \Gamma \left(-2.1911t+460.62,0.0011t+0.2658\right)\\ \ln\sigma_{s2}^{\max}\sim N\left(0.0023t+3.6126,\left(0.347t+0.1601\right)\times 10^{-3}\right) \end{array}\\ v_{3}=9\;m/s\;\begin{array}{l} \sigma_{\lim3}\sim \Gamma \left(-2.1692t+448.10,0.0011t+0.2733\right)\\ \ln\sigma_{s3}^{\max}\sim N\left(0.0023t+4.4360,\left(0.433t+0.0063\right)\times 10^{-3}\right) \end{array} \end{array}$$

**Figure 4:**
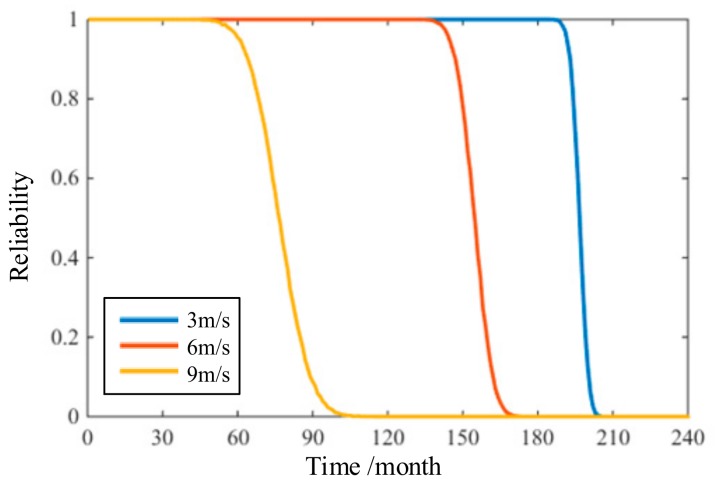
Time-variant reliability curves at different seawater velocities.

After calculating the reliability, the time-variant reliability curve of copper bending pipe can be obtained and is shown in Figure 4. It is obvious that the reliability began to fall from the 184th month and reached 0 at the 208th month in a seawater velocity of 3 m/s; the fall time lasted 24 months. The declining range of the reliability curve at 6 m/s was the 131th month to the 175th month and lasted for 44 months. The same range at 9 m/s was the 41th month to the 121th month and lasted for 80 months. Therefore, there is reason to believe that the greater the seawater velocity, the earlier the reliability of copper bending pipe begins to drop, and the longer the declining time is.

## 4. Discussion

### 4.1. Compared with Traditional Method

In practical applications, the replacement process of copper bending pipe is generally determined by traditional methods. However, due to the traditional method expressing the limit strength in the minimum value and the thickness of corrosion in the maximum value, this may result in the waste of the remaining life of the copper bending pipe and may be inaccurate. It can be considered that the copper bending pipe is reliable when the limit strength is larger than the maximum stress, and the reliability calculated by the traditional method is subject to the extreme distribution of 0–1. Since the stochastic degradation process proposed in this paper regards both limit strength and thickness of corrosion as random variables over time, and the reliability curves were obtained by solving the reliability at different times, it ensures the continuity and accuracy of the degradation process.

Therefore, making a comparison for this case, the relationship between thickness and maximum stress is also calculated by COMSOL, and the comparison results are shown in Table 5. At the seawater velocity of 3 m/s, the complete failure time predicted by the traditional method is twice as much as that by the method proposed here. Meanwhile, at the seawater velocities of 6 m/s and 9 m/s, for the copper bending pipe, the complete failure time predicted by the traditional method corresponding to the reliability calculated by the method proposed in this paper, respectively, are 0.9732 and 0.9986. The traditional method is greatly different from the method proposed in this paper.

### 4.2. Verifying the Accuracy of Two Methods by Maintenance Records

**Figure 5:**
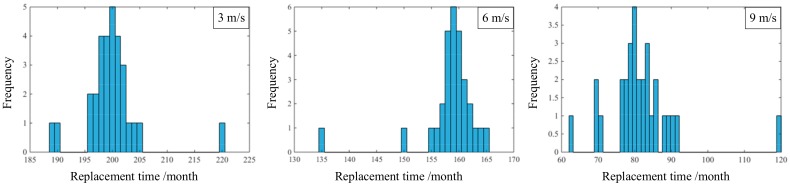
The histogram of replacement time.

In order to verify the accuracy of two methods, it is necessary to collect maintenance records of copper bending pipe. Figure 5 shows the maintenance records under actual working conditions (replaced until the leakage occurred).

The safety life of copper bending pipe is the time before a lot of failures occur [35]. According to the maintenance records, the safety life of copper bending pipe at 3 m/s, 6 m/s, and 9 m/s is 196 months, 155 months, and 76 months, respectively. The safety life from maintenance records corresponding to the reliability of the method proposed in this paper is 0.6036, 0.4848, and 0.5191, respectively. Furthermore, the safety life predicted by the traditional method at 3 m/s is too optimistic, while at 6 m/s and 9 m/s, is too pessimistic. Therefore, comparing these two methods, under the premise of 0.5 as a reliability indicator in the reliability model of this paper, the method proposed in this paper can evaluate the reliability of copper bending pipe more accurately.

In addition, the mathematical model used to describe the randomness and multiplicity of the degradation process under corrosion, considers both the performance degradation and the geometric degradation, and the use of simulation to obtain the stress model. Since randomness and multiplicity also exist in the degradation process of other materials, this mathematical model can also be used to describe them.

## 5. Conclusions

Based on the stochastic degradation process, this paper analyzes the time-variant reliability of copper bending pipe under seawater-active corrosion. According to the case study and discussion, the following conclusions can be drawn:

(1) During the degradation process, using the relationship of characteristic parameters over time to represent the stochastic degradation process, the randomness at any time can be easily and accurately described. Based on this, the stochastic degradation process of copper bending pipe is the PDF which contains a function over time, and the reliability curve can be calculated by interference theory.
(2) The life of copper bending pipe calculated by the method proposed in this paper and the life calculated by the traditional method were different by nearly two times. Comparing the maintenance records, it is more accurate and secure to obtain the replacement cycle of copper bending pipe by choosing 0.5 as the reliability indicator of the method in this paper.
(3) The mathematical model which was used to describe the degradation of copper bending pipe can also be used to model the degradation process of other materials due to the widespread occurrence of randomness and multiplicity.
(4) This paper proposes a kind of Monte Carlo method to solve the random distribution over time. At any time, by comparing the pseudorandom from two PDFs and recording the number of times that the limit strength is larger than the maximum stress, the reliability is the ratio of these records.

## Figures and Tables

**Table 1:** Experiment description.

Type	Parameters	Value
Experiment	Experiment period	24 months
	Experiment interval	3 months
Seawater	Density	1025 $\mathrm{kg}/m^{3}$
	Dynamic viscosity	0.8937 Pa·s
Copper material	Density	8960 $\mathrm{kg}/m^{3}$
	Young’s modulus	$1.1\times 10^{11}\;\mathrm{Pa}$
	Poisson’s ratio	0.35
Loads	Fluid velocity	3 m/s, 6 m/s, 9 m/s

**Table 2:** Characteristic parameters of limit strength.

No.	t/Month	α	β
1	3	464.3188	0.2638
2	6	458.8756	0.2661
3	9	451.8773	0.2695
4	12	446.1352	0.2722
5	15	440.3274	0.2750
6	18	436.2393	0.2768
7	21	429.5355	0.2803
8	24	413.1314	0.2903

**Table 3:** Characteristic parameters of thickness.

No.	t/Month	μ	$\sigma^{2}$
1	3	2.2976	0.0008
2	6	2.2872	0.0015
3	9	2.2827	0.0023
4	12	2.2763	0.0031
5	15	2.2748	0.0033
6	18	2.2653	0.0047
7	21	2.2569	0.0051
8	24	2.2513	0.0058

**Table 4:** Characteristic parameters of maximum stress.

No.	t/Month	μ	$\sigma^{2}$
1	3	2.2976	0.0008
2	6	2.2872	0.0015
3	9	2.2827	0.0023
4	12	2.2763	0.0031
5	15	2.2748	0.0033
6	18	2.2653	0.0047
7	21	2.2569	0.0051
8	24	2.2513	0.0058

**Table 5:** Comparison results of the two methods.

Flow Velocity	Type	Complete Failure	Corresponding Reliability in This Paper
3 m/s	Traditional	506 months	0
	This paper	208 months	-
6 m/s	Traditional	142 months	0.9732
	This paper	175 months	-
9 m/s	Traditional	45 months	0.9986
	This paper	121 months	-

**Figure A1:**
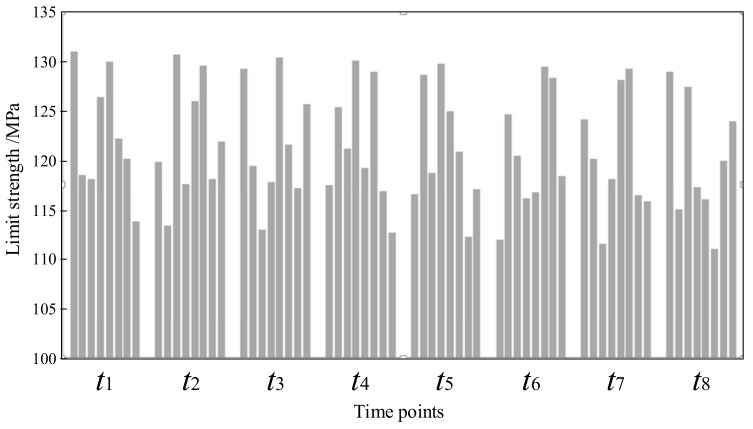
The distribution of limit strength at different time under 3 m/s.

**Figure A2:**
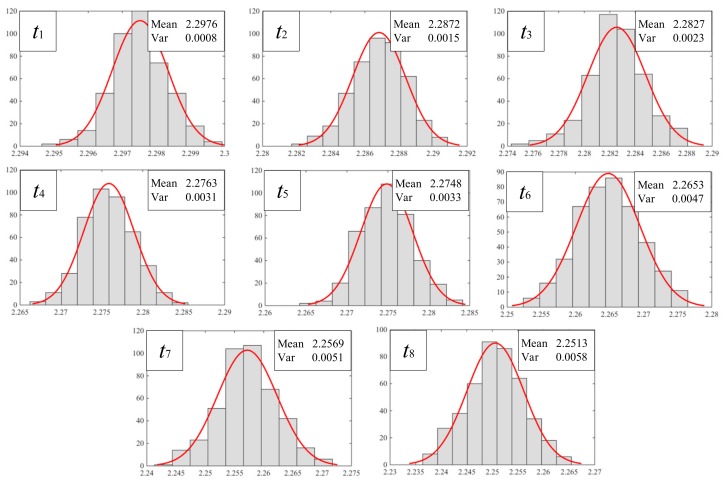
The distribution of thickness at different time under 3 m/s.
